# Spinal anesthesia: a safe alternative for high-risk pulmonary patients undergoing lumbar spine surgery

**DOI:** 10.1093/jscr/rjaf207

**Published:** 2025-04-06

**Authors:** Lennard M Wurm, Olaf Kniesel, Dominik Laue

**Affiliations:** Department of Traumatology and Reconstructive Surgery, Charité – Universitätsmedizin Berlin, Freie Universität Berlin, Humboldt-Universität zu Berlin and Berlin Institute of Health, 10117 Berlin, Germany; Medical Faculty of Heinrich-Heine University and University Hospital Düsseldorf, 40225 Düsseldorf, Germany; Department of Anesthesiology and Intensive Care Medicine, Charité – Universitätsmedizin Berlin, Freie Universität Berlin, Humboldt-Universität zu Berlin and Berlin Institute of Health, 10117 Berlin, Germany; Department of Traumatology and Reconstructive Surgery, Charité – Universitätsmedizin Berlin, Freie Universität Berlin, Humboldt-Universität zu Berlin and Berlin Institute of Health, 10117 Berlin, Germany

**Keywords:** spinal anesthesia, disc herniation, lumbar spine surgery

## Abstract

This case report details the successful management of a 58-year-old male patient with advanced chronic obstructive pulmonary disease and sarcoidosis, who required lumbar spine surgery for a cranial-translocated disc sequestration at the L4/5 level. Due to the patient’s significant pulmonary risk, spinal anesthesia (SPA) was selected to minimize the potential respiratory complications typically associated with general anesthesia. The procedure was successful, resulting in the complete alleviation of lumbosciatic pain without any notable complications. This case highlights the efficacy and safety of SPA in patients with high pulmonary risk, emphasizing its advantages such as reduced intraoperative blood loss, fewer postoperative complications, and enhanced postoperative recovery.

## Introduction

Patients with severe pulmonary comorbidities present significant challenges in the perioperative management of spinal surgeries. General anesthesia can exacerbate pulmonary conditions, leading to increased perioperative risk. Spinal anesthesia (SPA) offers an effective alternative by avoiding the need for intubation and mechanical ventilation, which can be particularly beneficial in patients with compromised respiratory function [1, 2]. The use of SPA in spinal surgeries is emerging as a viable option due to its associated benefits, including lower intraoperative blood loss, reduced pulmonary complications, and fewer cardiac ischemic incidents [3–5]. This report discusses the use of SPA in a patient with significant pulmonary impairment undergoing lumbar spine surgery, focusing on its efficacy and safety.

## Case description

A 58-year-old male, with a height of 166 cm and a weight of 70 kg, presented with severe, exacerbating lumbosciatic pain radiating to the right leg, predominantly affecting the L4 and L5 dermatomes. His medical history was notable for chronic obstructive pulmonary disease (COPD) Gold IV, sarcoidosis with pulmonary fibrosis (ED 18: 38% of predicted lung capacity), and methotrexate (MTX) therapy. Additionally, the patient had significant comorbidities including tricuspid valve insufficiency grade II with a history of pulmonary arterial hypertension, for which the last cardiological evaluation in November 2023 indicated no need for intervention and an overall normal pump function. He had a history of pancreatitis in 2021 and a 50 pack-year history of nicotine abuse. The patient also had non-insulin-dependent diabetes mellitus (NIDDM II) and required supplemental oxygen (1–2 L/min), with a SpO2 of 98% under oxygen therapy. His physical capacity was reduced (<4 METs). Blood pressure readings were 97/85 mmHg. The most recent pulmonary function test in March 2024 showed an EVCex of 43%, FEV1 of 38%, and EEVIEVC of 88%.

Physical examination revealed palpable tenderness over the lumbar vertebrae and paravertebral muscles, with positive Lasegue’s and Kernig signs indicating sciatic nerve involvement. A magnetic resonance imaging (MRI) conducted on 1 June 2024, revealed a cranial-translocated disc sequester at L4/5 on the right side as the source of the sciatic pain.

The patient underwent a minimal invasive sequestrectomy at L4/5 under SPA. The procedure was meticulously conducted under strict sterile conditions. SPA was administered at the L2/3 vertebral level following local anesthesia with 1% Lidocaine. A single puncture technique via 25G pencil point needle was used, with free-dripping cerebrospinal fluid confirming proper placement. A total of 2.8 ml of 0.5% bupivacaine was administered, achieving a sensory blockade up to the TH10 level without paresthesia or circulatory symptoms. The patient self-positioned into the prone position after SPA was applied, received 2 L/min oxygen via mask, and no sedation was given.

The surgical procedure was carried out through a 2 cm longitudinal mid line skin incision and a trans-tubular microscopic assisted nucleotomy and sequestrectomy. The intraoperative blood loss was estimated at ⁓15 ml. The interventional duration was 31 min without any complications. It was easy to communicate with the patient during the intervention and minimal patient movements did not affect the operation.

Postoperatively, the patient was closely monitored in a high-dependency unit with continuous oxygen supplementation and pulmonary physiotherapy. Pain management was effectively achieved through spinal analgesia and oral analgesics. Remarkably, the patient experienced complete resolution of lumbosciatic pain and significant improvement in motor and sensory function in the lower limbs. Follow-up after 1 month demonstrated substantial recovery, with the patient regaining independence in daily activities.

## Discussion

This case demonstrates the efficacy of SPA in managing high-risk pulmonary patients undergoing lumbar spine surgery. The choice of SPA minimized the risk of respiratory complications associated with general anesthesia. Literature supports the use of regional anesthesia in patients with severe pulmonary disease, as it can reduce the incidence of perioperative pulmonary complications [2]. SPA avoids the need for mechanical ventilation, which can be particularly beneficial for patients with compromised lung function [1]. In this case, the absence of respiratory complications postoperatively highlights the advantages of SPA in such high-risk patients. Furthermore, the literature indicates that SPA can offer better postoperative pain control and faster recovery times compared to general anesthesia [3].

The increasing use of SPA for spinal surgeries, including complex procedures such as lumbar fusions, indicates a paradigm shift in anesthetic management for these surgeries. Recent studies have shown that SPA can result in shorter operating room times, lower postoperative pain scores, and quicker ambulation compared to general anesthesia [4, 6].

In addition to the points raised, this case underscores the importance of multidisciplinary collaboration in optimizing outcomes for high-risk pulmonary patients. By engaging respiratory therapists, anesthesiologists, and spine surgeons in a concerted effort, we can tailor anesthesia plans to minimize perioperative complications [7]. A key consideration is the avoidance of intubation, as mechanical ventilation may exacerbate preexisting pulmonary pathology in patients with advanced COPD or restrictive lung disease. Furthermore, SPA allows for continuous patient feedback throughout the procedure, which can be advantageous if sudden neurological changes occur [8]. In this setting, sedation can be offered on a case-by-case basis, ensuring that respiratory drive remains uncompromised. Another notable benefit is the lower likelihood of postoperative delirium, as opioid and sedative use can be limited under regional techniques. The success of SPA in this patient was facilitated by careful hemodynamic monitoring, as well as prompt mobilization and effective pulmonary physiotherapy in the immediate postoperative phase. Although larger prospective trials are warranted to validate these findings, the positive outcome described here aligns with emerging literature that champions SPA as a feasible and beneficial strategy for spinal procedures in patients with compromised respiratory function. This paradigm shift ensures safer surgery and quicker recovery.

## Conclusion

SPA is a safe and effective alternative to general anesthesia for patients with severe pulmonary conditions undergoing lumbar spine surgery. Patient selection in terms of compliance and the ability to lie prone is crucial. Multidisciplinary collaboration and careful perioperative planning are essential for optimizing patient outcomes. This case underscores the potential for SPA to provide excellent pain control and recovery in high-risk patients, as evidenced by the complete resolution of lumbosciatic pain in this patient.

## Data Availability

Data generated during and/or analyzed during the current study are available from the corresponding author on reasonable request.
